# THE HEALTH TRAJECTORY OF OLDER IMMIGRANTS IN CANADA: FINDINGS FROM THE CANADIAN LONGITUDINAL STUDY ON AGING

**DOI:** 10.1093/geroni/igae098.4278

**Published:** 2024-12-31

**Authors:** Lun Li, Andrew Wister, Yeonjung Lee

**Affiliations:** MacEwan University, Edmonton, Alberta, Canada; Simon Fraser University, Vancouver, British Columbia, Canada; Chung-Ang University

## Abstract

In Canada, older immigrants constitute about one-third of aging Canadians, and the population size of older immigrants is projected to keep growing. The health and wellbeing of older immigrants are critical to Canada’s healthcare and social services systems. This study intends to examine the health trajectory of older immigrants in Canada over ten years (2011 to 2021) using three waves of data from the Canadian Longitudinal Study on Aging based on Linear Mixed Models. The data analysis unit contains a total of 21,480 older adults (aged 65 years and older), including 4,248 immigrants and 17,232 Canada-born. The results indicate that older immigrants report better physical health (fewer chronic diseases and better physical capability) than Canada-born older adults but experience a greater decline in physical health over ten years. When it comes to mental health, older immigrants and Canada-born older adults report similar levels of depression and loneliness, and the change of mental health is parallel over time. In addition, older immigrants report significantly lower levels of social wellbeing (e.g., social support and social participation), and a greater decline in perceived social support and social participation. The findings from this study enrich the “healthy immigrant effect” with older immigrants’ health situation in Canada over ten years. The findings also emphasize the need to support older immigrants in Canada to maintain social wellbeing and enhance the positive impacts of social determinants of health during the aging process.

